# Comorbidities, sociodemographic factors, and determinants of health on COVID-19 fatalities in the United States

**DOI:** 10.3389/fpubh.2022.993662

**Published:** 2022-11-03

**Authors:** Jacob Gerken, Demi Zapata, Daniel Kuivinen, Isain Zapata

**Affiliations:** Department of Biomedical Sciences, Rocky Vista University, Parker, CO, United States

**Keywords:** CCVI, sociodemographic factors, politics, county level, determinants of health

## Abstract

Previous studies have evaluated comorbidities and sociodemographic factors individually or by type but not comprehensively. This study aims to analyze the influence of a wide variety of factors in a single study to better understand the big picture of their effects on case-fatalities. This cross-sectional study used county-level comorbidities, social determinants of health such as income and race, measures of preventive healthcare, age, education level, average household size, population density, and political voting patterns were all evaluated on a national and regional basis. Analysis was performed through Generalized Additive Models and adjusted by the COVID-19 Community Vulnerability Index (CCVI). Effect estimates of COVID-19 fatality rates for risk factors such as comorbidities, sociodemographic factors and determinant of health. Factors associated with reducing COVID-19 fatality rates were mostly sociodemographic factors such as age, education and income, and preventive health measures. Obesity, minimal leisurely activity, binge drinking, and higher rates of individuals taking high blood pressure medication were associated with increased case fatality rate in a county. Political leaning influenced case case-fatality rates. Regional trends showed contrasting effects where larger household size was protective in the Midwest, yet harmful in Northeast. Notably, higher rates of respiratory comorbidities such as asthma and chronic obstructive pulmonary disease (COPD) diagnosis were associated with reduced case-fatality rates in the Northeast. Increased rates of chronic kidney disease (CKD) within counties were often the strongest predictor of increased case-fatality rates for several regions. Our findings highlight the importance of considering the full context when evaluating contributing factors to case-fatality rates. The spectrum of factors identified in this study must be analyzed in the context of one another and not in isolation.

## Introduction

The SARS-CoV-2 Virus, which causes COVID-19, has currently led to over 1,000,000 deaths (July, 2022) in the United States (1). The virus has ravaged not only the United States financially and put a stop to every in-person activity for the last 2 years (2), but has highlighted how embedded community traits affect the outcome of a whole community. In the end, it is characteristics like comorbidities and sociodemographic factors that play a defining role in determining a community's fatality outcome (3).

While it has been shown that a wide range of comorbidities has an impact COVID-19 outcomes; there has been a great effort to define the specific contributions of comorbidities in their impact on COVID-19 morbidity and mortality rates (4–7). Comorbidities such as hypertension (8), diabetes mellitus (9), chronic kidney disease (CKD) (10), chronic obstructive pulmonary disease (COPD) (11), and cardiovascular disease or coronary heart disease (CHD) (12) among others, have important repercussions on COVID-19 outcomes. However, there are also a variety of reasons for which to consider some sociodemographic factors as deleterious. COVID-19 outcomes are strongly influenced by risk behaviors and many of these behaviors are a direct result of environmental and socioeconomic circumstances that affect specific portions of the populations (13). Factors such as lacking healthcare insurance, being of a specific racial background (7, 14), or even voting patterns. Political voting patterns and affiliation have previously been linked to COVID-19 fatality in both the United States (15) and other countries (16, 17). While political differences may not be important on an individual level, political affiliation may influence societal behaviors such as vaccination, masking (18), or social distancing (19). These behaviors can directly affect COVID-19 outcomes, and are an important component of our analysis. Voting patterns in the 2020 presidential election are representative of behaviors that can affect COVID-19 outcomes. All these factors combined would point in a direction that suggests that differences in COVID-19 fatality rates are a potential outcome of embedded community characteristics.

Even though previous studies have examined factors influencing COVID-19 fatalities (4–6, 20), no study has performed a comprehensive analysis of all the aforementioned factors together to the same extent as our study. In our study, a multitude of key community indicators such as comorbidities, sociodemographic factors (including voting patterns), and determinants of health (preventive health screenings) have been examined to reflect trends and potential associations that can be compared against each other. Therefore, the objective of this study was to perform a comprehensive evaluation of comorbidities, sociodemographic factors, and determinants of health at a national level using county aggregated to define their association to COVID-19 case-fatalities. These patterns may allow us to alter the way communities handle public health crises, utilize public health interventions that could deflect harmful outcomes, and provide resources to communities in a timely manner based on their community characteristics.

## Methods

### Datasets

The focus of this ecological study was to evaluate regional trends of COVID-19 case-fatality rate compared to comorbidities and sociodemographic factors. This study was vetted and categorized as exempt by the Institutional Research Board. Our study utilized countywide data for each county in the entire continental United States and Hawaii with Alaska and Puerto Rico excluded from the analysis due to differences in their county data reporting. COVID-19 case-fatality rates were gathered from the COVID-19 Community Profile Report (21) for the January 2-8, 2021 week cutoff, this report included the COVID-19 Community Vulnerability Index (CCVI) (22). This cutoff week was selected because it allowed for the evaluation of the COVID-19 case fatality rate, without the influence of the vaccines or newer, more infectious strains. Rates of various comorbidities such as chronic obstructive pulmonary disease (COPD), hypertension, cancer, asthma, chronic heart disease (CHD), cholesterol, diabetes, chronic kidney disease (CKD), smoking, stroke, and obesity were obtained on a per-county basis from the CDC 2020 Population Level Analysis and Community Estimates (PLACES) project (23). Rates of poor mental health, binge drinking, lack of health insurance, time allocated to leisurely activity, and preventive care consisting of cervical cancer screening, routine doctor visits, dental visits, cholesterol screening, and routine mammography were obtained from the 2020 CDC PLACES Project, as well. Other variables such as average household size and population density for each county were acquired from the United States Census Bureau COVID-19 website (24). Latitude of each county was also included in the analysis and obtained from the United States Gazetteer Files (25) from the United States Census Bureau. The 2020 Presidential voting records of each county were obtained from the Harvard Dataverse (26). Racial makeup in each county was obtained from the 2020 decennial United States census (27), while income, age, and education level were retrieved from the 2019 American Community Survey 5-year estimates (28). A summary of all mean values per variable on a national level and by HHS region are presented in Supplementary Table 1.

### Statistical analysis

Data was evaluated for associations using a Generalized Additive Models (GAMs) approach. GAMs were chosen for this application for their versatility in addressing deviations from normality that limit Generalized Linear Models (GLMs) such as those that occur in proportional data (29, 30). More specifically, when values approach the limits of the scales (such as percentages), these models take advantage of unspecific (non-parametric) functions or splines that are linked to the predictor (31). COVID-19 case fatality rates per 100 k people were set as the dependent variable while each comorbidity, sociodemographic and health determinant factor was set as an independent variable. All values are proportional data. All models were adjusted using CCVI (22) which normalized the data for inherent inequity on a county per county basis (32). This ecological study uses individual counties as the experimental unit. All analyses were evaluated twice, once nationally and once regionally. Analyzing using two different modes, allowed for us to identify national and regional patterns (US HHS regions). Independent variables were introduced into the model using smoothing splines starting with three degrees of freedom. Models assumed Gaussian residual distributions. All analyses were performed using PROC GAM in SAS/STAT v.9.4 (SAS Inc., Cary, NC). Risk ratios were estimated with confidence intervals and the coefficients sign determined effect directions. GAMs estimates can be interpreted in a similar fashion a parametric GLMs. Therefore, negative coefficients indicated a reduction in COVID-19 case-fatality rates while positive coefficients indicated an increase in case-fatalities. Coefficient standardization was done with a normally distributed Z-score transformation. All associations presented were tested using two-tailed tests. Regional pattern models were performed independently to identify the top contributors—negatively and positively associated. Even with 99% confidence intervals, all tests were declared significant at a Bonferroni threshold. Regional pattern top contributors that did not reach the Bonferroni threshold are indicated in the figures.

## Results

### National level trends

**Figure 1:**
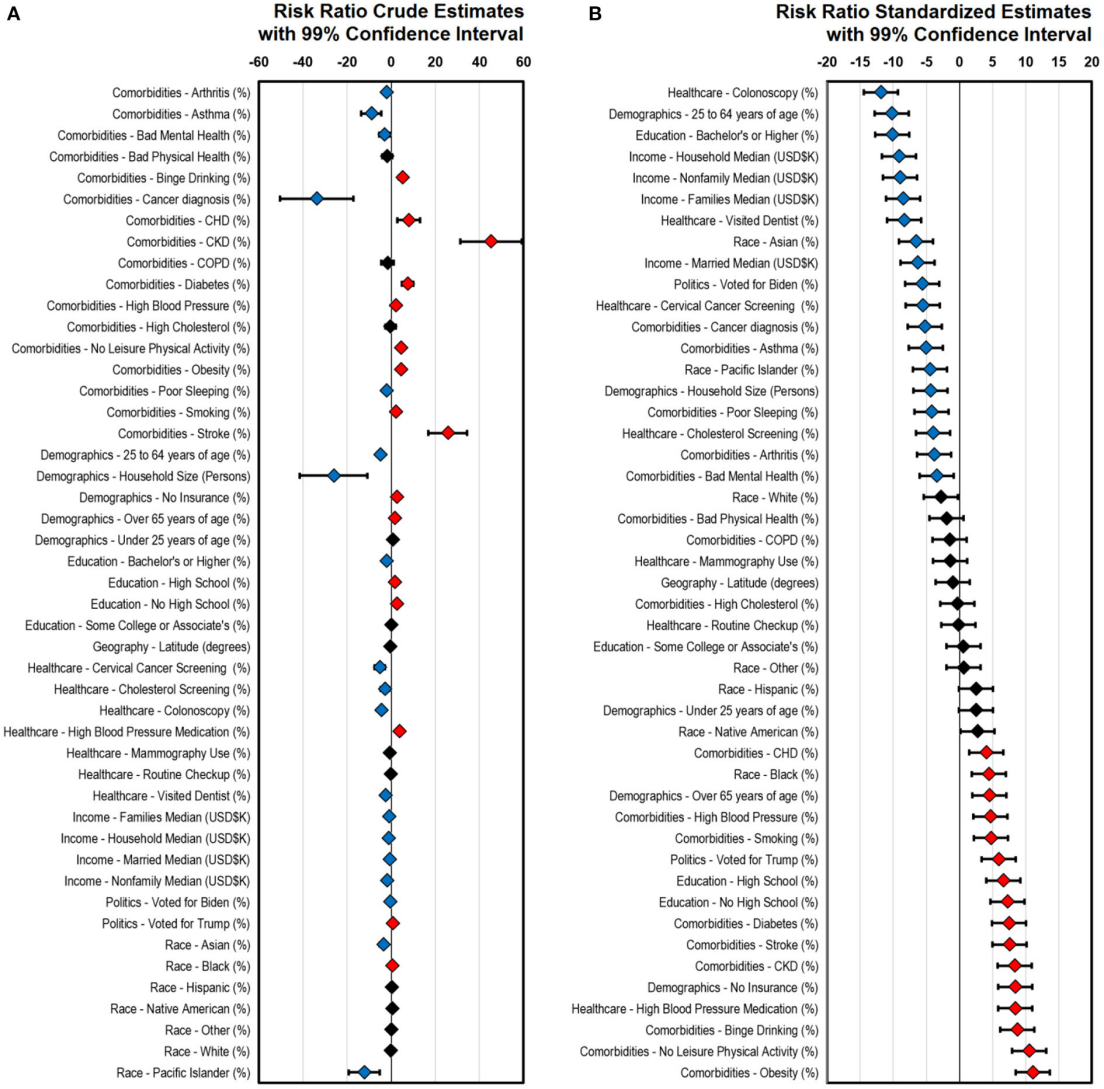
Comorbidity, sociodemographic and determinants of health associations to COVID-19 case-fatalities. All models are CCVI adjusted. **(A)** Risk ratio crude estimates. **(B)** Risk ration standardized estimates (ordered). Blue diamonds indicate case-fatality reduction, red diamonds indicate case-fatality increase, and Black diamonds indicate no association. A total of 3140 counties were included in the study. Even when 99% CI are presented, association are declared significant at a Bonferroni adjusted threshold (47 tests P_adj_ ≤ 1.06E-03). COPD, Chronic Obstructive Pulmonary Disease; CKD, Chronic Kidney Disease; CHD, Coronary Heart Disease.

Data from 3140 counties was included in our analysis. Our GAM approach examined the data for associations to case-fatalities, all these values were adjusted to CCVI to normalize the inherent differences a crude risk ratio would estimate, and the risk ratio standardized estimates are presented in Figure 1. Crude estimates showcase the extent of the association without considering their spread while standardized estimates adjust the extent of the association to the spread. Crude estimate findings (Figure 1A) revealed that comorbidities above sociodemographic factors have the largest effects associated with case-fatalities; however, these associations can go in both positive and negative directions. A diagnosis of cancer provided the largest effect decreasing COVID-19 case-fatalities while CKD and stroke had the largest effects increasing them. Similarly, asthma (decreased risk), CHD (increased risk) and diabetes (increased risk) displayed less extensive effects. Household size was the largest significant sociodemographic factor in positive outcomes of COVID-19 with an effect in a range comparable to relevant comorbidities. Other demographic effects such as age and education displayed significant associations that reduced or increased case-fatality risk. Populations with higher educational achievements displayed significantly reduced case-fatality rates. Increased income always displayed a protective effect. Political preference was significantly associated with case-fatalities such as voting for Biden reduced case-fatalities while voting for Trump had the opposite effect. Racial and ethnic backgrounds were only associated with COVID-19 case–fatalities for Pacific Islanders, Asian and Black groups. Determinants of health such as cervical cancer screening and people using high blood pressure medication also showed mixed direction associations. Cervical cancer screening had the largest case-fatality reducing effect from this category while the people using high blood pressure medication had the largest opposite effect. Standardized estimates (Figure 1B) show a different perspective and allow for comparisons across factors as they are standardized. In this case, a routine colonoscopy procedure was found to be the largest protecting effect against COVID-19 case-fatality followed by a combination of sociodemographic factors such as age, education, and income. On the other side of the spectrum, obesity had the largest negative impact deleterious outcome in COVID-19 patients followed by having no leisure physical activity, binge drinking and higher proportions of people taking high blood pressure medication in a specific county.

### Regional trends

**Figure 2:**
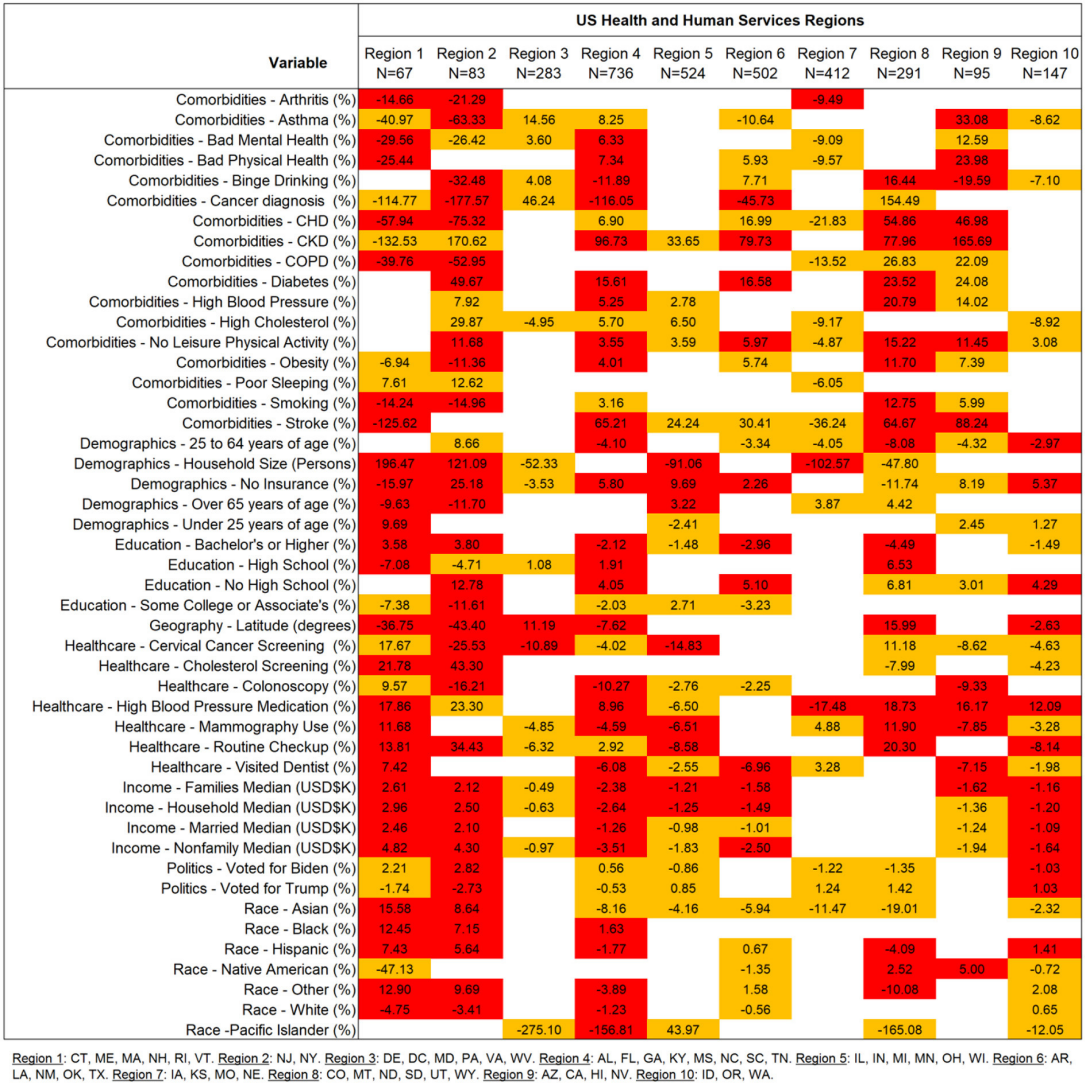
Regional risk ratio estimates for comorbidity, sociodemographic and determinants of health in association to COVID-19 case-fatalities. All models were performed independently by region and are adjusted to CCVI. Sample sizes correspond to the number of counties in the analysis for each region. Estimates signs indicate effect direction. Red boxes are significant to a Bonferroni level (470 tests P_adj_ ≤ 1.06E-04). Orange boxes are significant to a 95% confidence level. COPD, Chronic Obstructive Pulmonary Disease; CKD, Chronic Kidney Disease, CHD, Coronary Heart Disease.

**Figure 3:**
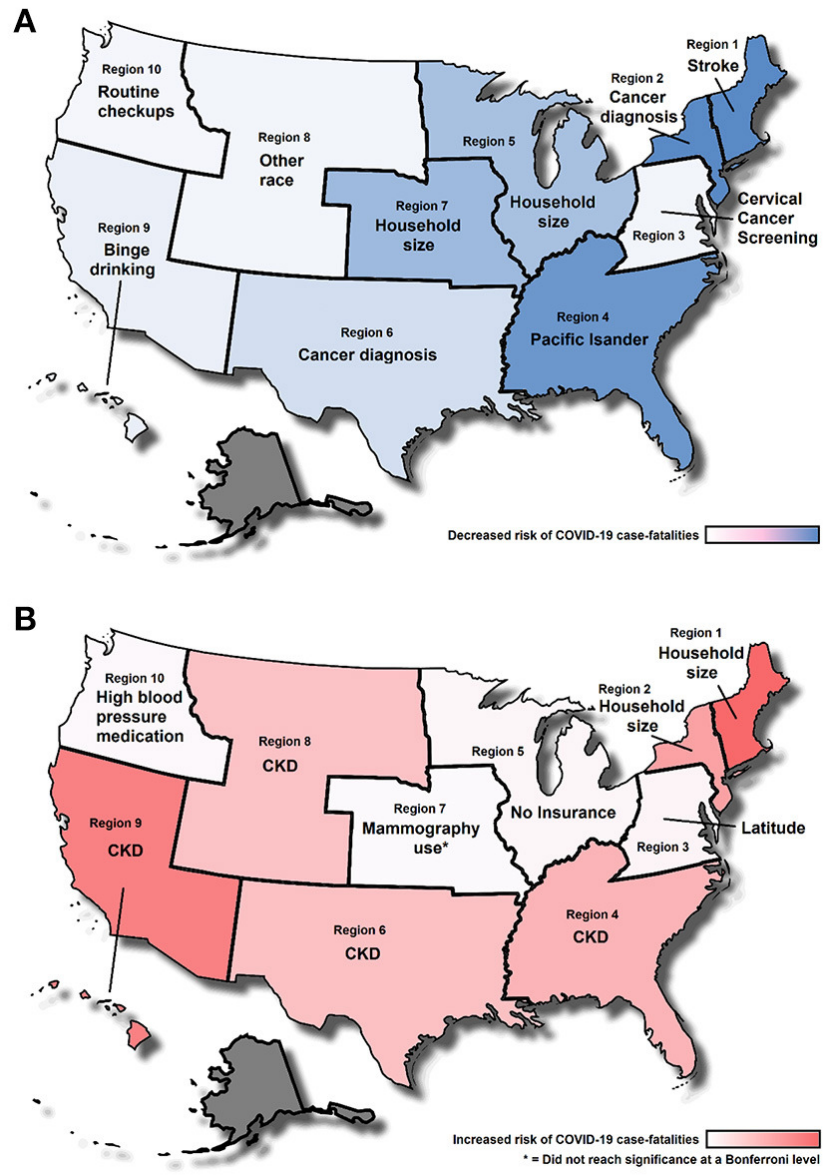
Top county level factors associated to COVID-19 case-fatalities for each United States Department of Health and Human Services regions. **(A)** Top factors associated with reduced case fatalities. **(B)** Top factors associated with increased case-fatalities. Shading indicates the effect size across regions (adjusted by COVID-19 Community Vulnerability Index, CCVI). Alaska was excluded from the analysis because of differences in their county level reporting. CKD, Chronic Kidney Disease.

The main analysis was also replicated independently within each of the ten US Health and Human Services defined regions. These models were also adjusted by CCVI. Risk ratio effect estimates for the ten regions are displayed in Figure 2. These analyses detected a wide array of effects that in some cases go in opposite directions across all regions. No single factor was consistently associated for all regions suggesting that regional associations are not generalizable. These regional assessments all have different sample sizes that are based on the number of counties within each state. These can range from 67 (Region 1) to 736 (Region 4); however, this discrepancy did not affect the capacity of each regional analysis to detect associations at a Bonferroni level (adjusted for 470 tests across all sets). The top variables reducing and increasing COVID-19 case-fatalities for each region are presented in Figure 3. The map in Figure 3A shows that the strongest protective regional effects were observed toward the east of the country where Stroke and Cancer Diagnosis were highly protective in the Northeast regions (Regions 1 and 2) and being of Pacific Islander descent was protective in the Southeast United States (Region 4). The Midwest displayed some moderate protective effects where household size was the top reducing factor in two regions (Region 5 and 7). Western regions displayed smaller COVID-19 case-fatality protective effects. Regions in the Western United States displayed smaller effect sizes in comparisons to regions in the South or Midwest. Lastly, Figure 3B shows variables that most significantly contributed to increased COVID-19 case-fatalities. CKD was the most prevalent comorbidity across several regions (Regions 4, 6, 8 and 9). Among other findings, household size had a negative impact on COVID-19 outcomes specifically in the Northeast regions (Regions 1 and 2). As previously mentioned, some of the top variables displayed opposing effects which suggested that the interpretation must be done in context with the specific characteristics of that region, and interpretations cannot be generalized to others.

## Discussion

The objective of this study was to perform a comprehensive evaluation of comorbidities, sociodemographic factors, and determinants of health at a national level using county aggregated to define their association to COVID-19 case-fatalities. Previous studies have evaluated the influence of various socioeconomic factors (7, 20, 33) and comorbidities (4–6) on COVID-19 case-fatality rate; however, these analyses do not pair together their findings to be comparable with each other. Our study evaluates COVID-19 fatality rates from a wide timeframe, without potential influence from vaccines reducing case-fatality rate and the addition of major COVID-19 variants. Our study builds on the efforts of previous studies by presenting together a wide array of variables that describe community characteristics. It is necessary to emphasize that the associations between these factors and case-fatalities is not necessarily or entirely causative. All comorbidities, sociodemographic and determinants of health variables presented describe characteristics of the population that are not isolated or independent and are antecedent of any causality. Therefore, any inference that could be attributed to each factor evaluated must always be provided with context as a community indicator as they are all dependent or interconnected on each other, examples of this are binge drinking, mammography and visits to the dentist rates, which are likely indirectly describing a characteristic of the community. In general, all these variables must be interpreted in a continuum of causality that can vary across regions depending on the context.

### Comorbidities

Chronic kidney disease rates were the strongest predictor of increase COVID-19 case-fatality in several US regions. This relationship is likely to be predominantly causal due to people with this condition being medically vulnerable (34–37). Other comorbidities followed a similar trend such as higher rates of hypertension, diabetes, stroke, and smoking being associated with an increased case-fatality rate in a specific county. This finding aligns with other studies (5, 38, 39) linking increased rates of comorbidities to poorer COVID-19 outcomes. Even though comorbidities were most often associated with worse COVID-19 outcomes, stroke and cancer diagnosis were linked to reduced case-fatality rates in the Northeast region. We speculate that a potential explanation for this relationship is possible more frequent mask usage (40) and more precautions taken by this group of people (41). Northeastern states had mask adherence rates > 75% during the latter part of 2020 (40) with usage potentially diminishing the influence of comorbidities such asthma and COPD on COVID-19 case-fatality rate. Those with asthma and COPD in these communities were maybe more likely to wear a mask, further reducing their chance of acquiring and succumbing to COVID-19.

### Sociodemographic factors

Household size was identified early on to be a risk factor for COVID-19 transmission (42). Our results have shown conflicting effects when viewing this factor across regions. Household size is a risk factor in the Northeast but has a protective effect in the Midwest. The difference between these regions is likely related to the specific context of living conditions. Although the mean household size for regions 1 and 2 is not far from the mean household size for regions 5 and 7, with 2.48 and 2.41 respectively, this difference may capture differences in housing quality and composition (43). This may also be indirectly identifying behavioral factors that are not obvious but can be implied such as house proximity (higher in cities) which can affect the capacity of self-isolation. Population density could partially be influenced by household size, which has shown to have an impact on transmission (44). Northeast states have higher population densities when compared to midwestern states. In summary, living conditions, housing quality and composition, and population density are all important components that define the impact of household size on case fatality rate. Generally, higher income was also associated with decreased COVID-19 case fatality rates. There could be a multitude of reasons why a higher income may be beneficial. This could include not being classified as an essential worker leading to being more likely to take time off or work from home (45), or even being able to live outside high-density population and compact housing areas (7, 46). Income is often reflective and associated with racial discrepancies in COVID-19 outcomes (33, 47, 48). In a study examining neighborhood median income and COVID-19, when examining the neighborhoods, Black populations more often lived in neighborhoods with a significantly lower median income ($35,000) whereas White populations more often lived in wealthier neighborhoods ($63,000). Communities with lower median incomes are more often Medicaid patients and have COVID-19 complications that require invasive tactics such as mechanical ventilation (49). Income inequalities have been strong predictors of higher case numbers and fatalities throughout this pandemic (20).

### Political affiliation

The context that defines the influence of social dynamics on COVID-19 is complicated. Political affiliation has been repeatedly evaluated as a potential factor influencing the pandemic's mortality (19, 50) *in prior* pandemics, with Republican party affiliation associated with decreased influenza vaccinations and Democratic party affiliation associated with increased vaccinations (51, 52). The politicization of pandemic response has continued into the COVID-19 pandemic, with behaviors such as masking, social distancing, and vaccination being often divided along party lines (17, 19). Some studies have shown a decrease in pandemic preventive health measures among Republicans while there has been increased adherence to public health recommendations among Democrats (53, 54). Similarly, Republicans have been shown to have lower COVID-19 risk perception (55, 56), when compared to other political parties which may influence their likelihood of contracting COVID-19. The behaviors exhibited by each political party may influence the results of this study. Our study showed that voting for Joseph Biden in the 2020 presidential election was mildly associated with decreased case-fatality rate while voting for Donald Trump was associated with increased rate. With Democrats being shown to be more likely to adhere to public health guidelines, they may be less likely to acquire and perish from COVID-19 while the inverse is true for Republicans. In December 2020, states with Republican governors had higher rates of cases, deaths, and positive tests than states with Democrat governors (57). This trend is evident in a similar approach using national data presented by NPR in collaboration with researchers at John's Hopkins University where it was shown that voting Republican also had a deleterious effect (58). Rural and urban differences have been shown to play a major role in case-fatality rate as well with rural counties having a higher case-fatality ratio than urban counties (4). Rural voters are more likely to vote Republican (59) and therefore, the influence of politics in our findings may also be capturing geographical differences. Rural areas tend to have worse health outcomes in general and have significantly less access to care compared to their urban counterparts (60). These disparities add to the likelihood of developing comorbidities and ultimately, poorer COVID-19 outcomes. The link between political affiliation and COVID-19 case fatality rate is far more complex than the individual candidates that people of a county voted for. Political affiliation in our study is an indicator of underlying sociodemographic, health, and psychological trends that are more causative rather than associative.

## Limitations

Our study utilized aggregate data on a per-county basis instead of individual patient data; therefore, it is not possible to evaluate factors that contribute to COVID-19 case-fatality on a per case fashion which could help avoid any erroneous generalizations of specific regions. Another limitation of using county level data is that there is significant variability in the size and number of counties across the United States. Some counties may have only a few hundred people, while other counties may have a few million and this may lead, to some extent, representation bias.

### Future prospective

Our study findings support a notion where all comorbidities, sociodemographic and determinants of health variables describe characteristics of the population; these characteristics are not isolated or independent but may share their etiology. For this reason, we believe our research can help inform future directions in public health including the evaluation of individual community factors that contribute directly to illness outcomes. Despite the application of our findings to public health, it is difficult to apply our findings to clinical practice recommendations due to the interdependent nature of the variables we evaluated. A future prospective should attempt to incorporate the factors identified into the population inclusion criteria of follow up approaches. In general, our study allows us to recommend expanding the list of confounders traditionally used in studies. These expanded confounders come in two main types: first, well defined groups at high risk (those with CKD, COPD or other prominent comorbidities) and second, less well defined groups with predisposition (those living in specific housing conditions, environments, or with specific political leanings). These factors are non-traditionally considered but have an important contribution to outcomes.

## Conclusions

Our study identified several unique regionally dependent and independent relationships that highlighted the various factors that might influence COVID-19. Like other studies, we determined that comorbidities and demographic factors together are strong drivers of COVID-19 case fatalities. However, our study presents an assessment that puts them side to side for direct comparison. Our study highlights how any association is often dependent on the regional context. For example, household size in the Northeastern region of the United States was associated with more fatalities, while larger household size in the Midwest regions had a protective effect. Political voting patterns were also indicative of underlying healthcare patterns, with overall reduced case fatality rates in Democratic voting counties compared to increased fatality rates in Republican voting counties. The trends we identified in our study emphasize the importance of interpreting each factor in the context of other variables instead of in isolation.

## Data availability statement

The original contributions presented in the study are included in the article/Supplementary material, further inquiries can be directed to the corresponding author.

## Author contributions

JG: conceptualization, methodology, data curation, investigation, and writing of original draft. DZ and DK: conceptualization, methodology, investigation, and writing of original draft. IZ: conceptualization, methodology, investigation, formal analysis, visualization, supervision, writing and editing of final draft. All authors approved the final manuscript as submitted and agree to be accountable for all aspects of the work.

## Conflict of interest

The authors declare that the research was conducted in the absence of any commercial or financial relationships that could be construed as a potential conflict of interest.

## Publisher's note

All claims expressed in this article are solely those of the authors and do not necessarily represent those of their affiliated organizations, or those of the publisher, the editors and the reviewers. Any product that may be evaluated in this article, or claim that may be made by its manufacturer, is not guaranteed or endorsed by the publisher.
